# A biological perspective toward the interaction of theranostic nanoparticles with the bloodstream – what needs to be considered?

**DOI:** 10.3389/fchem.2015.00007

**Published:** 2015-02-10

**Authors:** Martin J. D. Clift, Jean-François Dechézelles, Barbara Rothen-Rutishauser, Alke Petri-Fink

**Affiliations:** ^1^BioNanomaterials, Adolphe Merkle Institute, University of FribourgFribourg, Switzerland; ^2^Department of Chemistry, University of FribourgFribourg, Switzerland

**Keywords:** blood, bloostream, nanomedicine, red blood cells, white blood cells, platelets, *in vitro*

Nanomedicine intends to create and further use novel materials at the nanoscale in order to provide an improvement upon current medical applications for human healthcare (ESF, 2005; Etheridge et al., 2013). In line with the advances made within nanotechnology since the late twentieth century (Mamalis, 2007) nanomedicine has received heightened attention due to its potential advantages, most notably within (cancer) theranostics (Muthu et al., 2014). The field of theranostics aims to utilize the physico-chemical characteristics of nanosized materials in order to intensify the effectiveness in diagnosing and treating diseases at the molecular level (Kim et al., 2013). Such a perspective is notably paramount for cancer types that are difficult to identify as well as apply therapy toward (e.g., secondary cancer) (Muthu et al., 2014).

Despite the well documented and proposed benefits of therapeutics in the nano-size range (Krol et al., 2013), for the majority of nanoparticles (NPs) [defined as “*a nano-object with all three dimensions in the nanoscale (1–100 nm)*″ (BSI, 2007; ISO 27687, 2008)], the ability to merge the expansive divide between developing a significant advancement within material science and creating a biologically relevant therapeutic has proven to be a highly non-trivial task. One important reason for this is the relatively limited specific understanding of the biological interaction of therapeutic NPs following their administration into the human body and their subsequent delivery to the target site (e.g., tumor) (Capco and Chen, 2014). The objective of this opinion article therefore, is to provide a biological perspective upon what must be considered in the development of theranostic NPs.

## Where should focus be given?

For biologically effective theranostic NPs, determining their dispersity, biocompatibility and biostability within different biological environments is imperative. For this, an understanding of the dynamic interaction between NPs with liquid and cellular systems as well as their subsequent biological impact must be gained. This outlook is not straight-forward and requires an intensive, multi-interdisciplinary research focus with cross-talk/feedback loops between the material scientists developing the materials and the biologists/clinicians wishing to study/apply them.

Initially, from a material perspective, there are an abundance of complex hurdles that must be overcome when developing any proposed nanotheranostic (Petros and DeSimone, 2010). Whether the NPs are manufactured for use as a treatment e.g., degenerative disease states (e.g., Alzheimer disease) (Liu et al., 2005), infectious diseases (e.g., hepatitis B) (Li et al., 2010) cancer (McMillan et al., 2014), or as a diagnostic tool (Niemirowicz et al., 2012), a systematic chemistry approach must be used (Davis et al., 2008). Whilst the specific shape of the NPs is of extreme interest regarding their efficiency as a theranostic agent (Liu et al., 2012), it is the precise material applied that is important, as well as the surface layer and the subsequent surface attachment of therapeutic agents and additional molecules (e.g., fluorophores, receptor-targetting moieties) to the modality (Petros and DeSimone, 2010), while keeping within the nano-size range. Additionally, determining their dispersity (i.e., colloidal stability) and biostability can also be laborious and problematic (Petros and DeSimone, 2010). Although these issues are not trivial, once the NP is engineered and ready for use, one of the main, biologically-based obstacles is to determine the ease of directing this modality to the site of interest within the human body without causing any undesirable effects (e.g., recognition and/or clearance by the immune system).

Successful targeting of theranostic NPs is an onerous concept (Nicolaides et al., 2014), and is commonly overlooked in favor of immediately focussing upon the effectiveness of the theranostic agent upon the specific target site (i.e., cancer cells for cancer therapeutics) (Xie et al., 2011). For example, a plethora of studies have been published which have shown the effectiveness of theranostic NPs in either the delivery of a drug to cells (Najafi et al., 2014), or destructing cancer cells with or without external stimuli (e.g., light, magnetic field) (Hayashi et al., 2014). Naturally, this approach is of extreme importance, and absolutely vital toward the development of any theranostic based NPs. However, the precise effective nature (i.e., efficacy) of the NPs upon the chosen target site can be considered as inextricably linked to the efficient transport of NPs from their administration site into the human body to the specifically chosen target site (Davis et al., 2008). Therefore it is essential that the interpretation of the effectiveness of theranostic NPs directly upon the target site is considered after gaining a controlled understanding of the biological impact upon the NPs following their transport through different biological environments to said target site.

Thus, in order to fully elucidate the impact of the transport processes following administration of theranostic NPs until reaching their target site, comprehension as to what biological entities interact with the NPs, how their physico-chemical characteristics may be altered over time and from interaction with different environments, as well as subsequently how these potential adaptations might affect the effectiveness of the NPs when they engage with the intended site of interest is decidedly necessary. Focus upon these key aspects would further enable the enhanced development of theranostic NPs from a materials' perspective, allowing them to be optimized for maximal benefit toward their proposed application. Through this approach, significant improvement to the efficacy of the NPs to the target site would be obtained concomitantly. Nonetheless, which route of transport toward the target site of the applied theranostic NPs should be studied first?

## Approaching the problem

**Figure 1:**
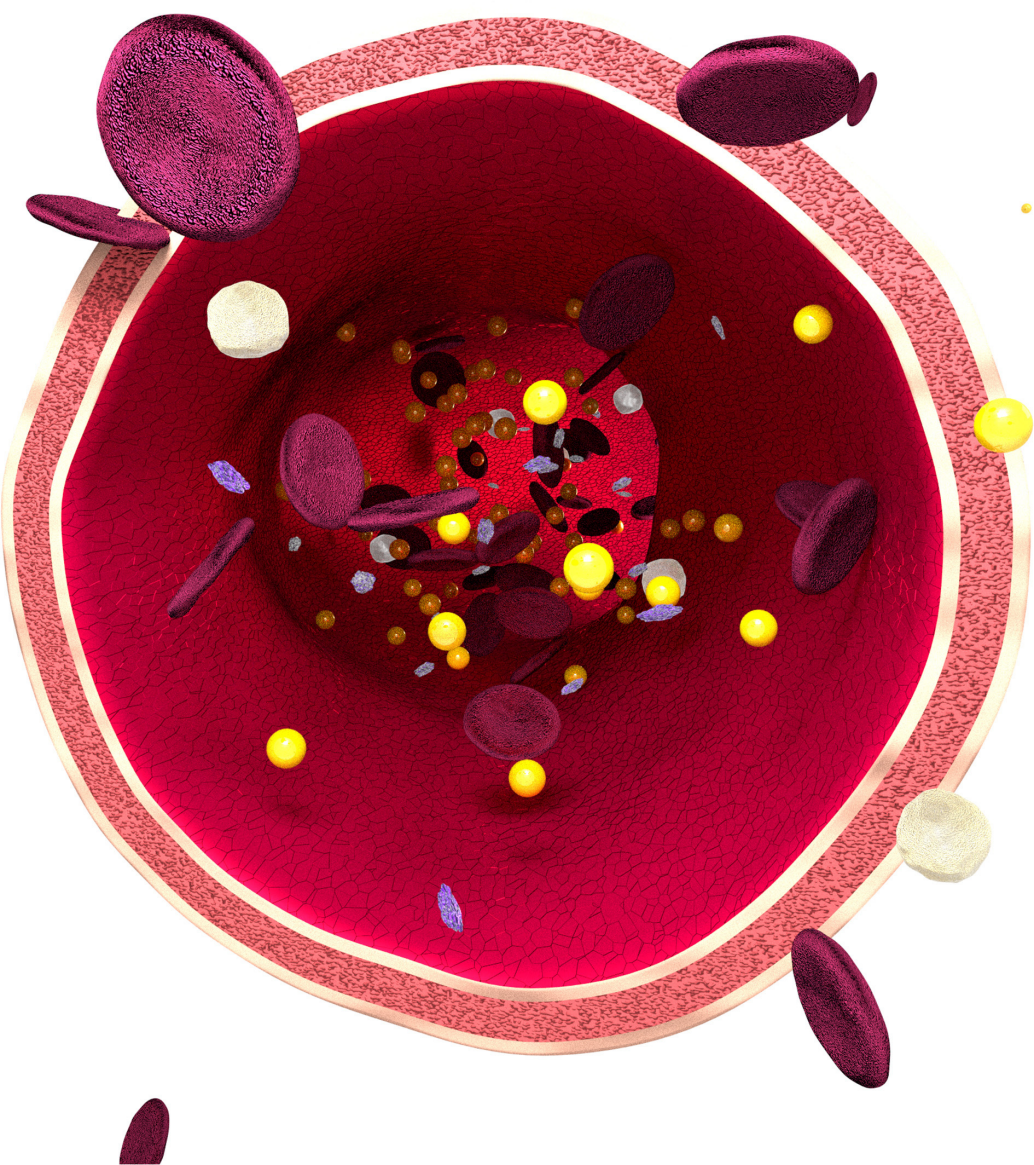
**Schematic of a human blood vessel, representative of the human bloodstream**. Image shows the three main cell types, (i) erythrocytes, also known as red blood cells (RBCs) (*represented as flat, disc-shaped red cells*), which contain hemoglobin (an iron-containing biomolecule responsible for oxygen (O_2_) binding), (ii) leukocytes (i.e., white blood cells) (*represented as white, round cells*) and (iii) thrombocytes (i.e., platelets) (*represented as small purple cells*), the essential cell type that allows for blood clotting (Abbas and Lichtman, 2003). The human bloodstream is responsible for the circulation of nutrients (i.e., amino acids), O_2_ and hormones, in addition to the removal of metabolic waste (e.g., carbon dioxide) (Abbas and Lichtman, 2003). It assists in regulating body temperature and pH, and further engages in the fighting of disease states. All of these functions contribute toward the essential maintenance of the homeostasis of the human body. In addition the human bloodstream is suspended within a protein matrix, abundant in albumin, known as plasma (i.e., blood serum together with fibrinogens) acting under the influence of non-classical hydrodynamic flow, known as haemodynamics (Abbas and Lichtman, 2003). The suitability of this holistic environment upon nanoparticles (*represented as gold spheres*) for theranostic applications is currently limited.

The main administration route for most theranostic-based NPs is *via* intravenous injection (Nichols and Bae, 2012). Thus, the initial biological environment that these theranostic-based NPs will encounter is the complex cellular and molecular milieu of the human blood circulation (as described in Figure 1). Thus, foremost direction toward understanding its impact upon theranostic NPs is paramount.

To systematically study the impact of the bloodstream upon thernostic NPs, *in vivo* (i.e., rodents) assessment would rapidly determine the efficacy of NPs formulated for theranostics. Yet, despite encompassing a “whole-body” scenario, it would not provide species specificity, which would be necessary for the inevitable application of NPs as theranostic agents. Primates would therefore be ideal, as used in the study by Ye et al. (2012), who showed the applicability of quantum dots as useful theranostic tools. However, neither *in vivo* strategy would provide the basis for a systematic study as to how NPs may interact with their numerous local environments (i.e., within the bloodstream) prior to arriving at their intended target site in the human body. By adopting an *in vitro* perspective however, it would enable a controlled outlook toward studying the impact of each biological constituent of the human bloodstream upon the chosen theranostic NPs. Difficulties in this approach arise however, since it would only allow for monoculture, or, at the most, co-culture systems to be used to conduct such investigations. Although advanced *in vitro* systems concerning the bloodstream and its constituent parts are being established, such as the advanced platelet model system recently reported by Thon et al. (2014), a finite model system that mimics the bloodstream is currently lacking. Therefore, currently, to comprehend how biological environments, such as the bloodstream, may impact upon the effectiveness of theranostic NPs a combined *in vitro* and *in vivo* approach should be integrated as a vital component in the development of theranostic NPs.

On an additional note, it is prudent to note that such a systematic study of any therapeutic NPs from the specific exposure site, via the potential transport route to the target site should be performed in order to gauge their potential effectiveness following administration. In this regard, it is also relevant to highlight that a series of other exposure routes, including ingestion, cutaneous and inhalation (Melancon et al., 2012), the latter for which theranostic applications are being derived (Pison et al., 2006), also pose a potential access route for NPs into the blood circulation *via* translocation across cellular barriers (Kreyling et al., 2012). Furthermore, the use of NPs to coat implants (i.e., for antimicrobial purposes) has recently increased (Kempe et al., 2010), and therefore it is possible that these could further concentrate the NPs gaining access into the human bloodstream, also *via* barrier cell translocation. Yet, the presence of NPs within the bloodstream from these exposure routes represents a secondary, non-specific exposure scenario and relates to a risk perspective. Whilst risk assessment is not the purpose of this article, it is worth to highlight that this issue has received limited attention to date, and requires further, in-depth investigation which could advantageously coincide with the advancement of NPs for nanomedicine-based applications (i.e., understanding their biocompatibility).

## Moving forward

Due to the lack of an advanced *in vitro* model system, as previously highlighted, determining the role of each component of the bloodstream as to its potential impact upon theranostic NPs is imperative to their overall development. However which constituents are important?

Most notably, the immediate and abundant adherence of proteins (as well as lipids) to the surface of any theranostic NPs entering the bloodstream (Lynch et al., 2006) can create a possible issue towards the surface molecules attached for a specific therapeutic purpose (i.e., receptor-binding sequence), as well as a loss in colloidal stability due to aggregation (Hirsch et al., 2014). Although NPs with varying physico-chemical characteristics can be manipulated for nanotheranostics, it has become abundantly apparent that similar proteins are consistently found upon the surface of NPs independent of their surface coating/charge (Hirsch et al., 2013). Whilst this is a dynamic process upon the surface of NPs, there remains a hard protein layer on top of the NPs at all times, thus posing a significant issue to material scientists. Yet, if coated with abundant proteins, these can engage with the epitopes on the immune cells, and so it is difficult to decipher if the steric repulsive barrier of a polymer shell would still remain effective enough to prevent uptake by these phagocytic cells, or not. Although, if internalized by the immune system, will they be processed and potentially exocytosed by these cell types, and exhibit the same properties prior to their administration? What the physico-chemical state of the NPs is following this interaction is currently unknown, and requires in-depth investigation. If however, the immune system does not recognize the NPs, then there is a heightened possibility that they could pass, unimpeded into erythrocytes (Rothen-Rutishauser et al., 2006). The impact that this cellular interaction may have upon the NPs is relatively unknown. Although if the NPs become present within these cell types, circulation time (of the NPs) will most likely increase, perhaps rendering them ineffective and/or aggregating within the bloodstream with potential adverse/fatal consequences in the long-term. In addition to these cellular/molecule based issues, the effect of the injection process (e.g., pressure, flow-rate, pH and temperature changes) upon the physico-chemical characteristics of the NPs *via* their administration route must also be conceived. Therefore, increased research strategies must be directed toward this approach to achieve the successful development of theranostic NPs.

## Overall perspective

Due to their inevitable administration to the human body via intravenous injection, understanding of the interaction of theranostic NPs with the complex biological environment of the bloodstream is vital in regards to their development. The knowledge created from this approach could enable key understanding to be gained as to the ability for the NPs to withstand the confines of this local environment. Furthermore, it will provide imperative insight into their ability to effectively perform the task they were engineered to achieve (e.g., drug delivery). Since following this approach the NPs will most likely require further manipulation regarding their physical and chemical characteristics, in order to achieve this outlook an enhanced, multi-interdisciplinary approach must be adopted. By combining the expertise of a variety of disciplines it will enable the advancement of systematic studies of the physical and chemical state of the NPs based on the impact observed when NPs are present within the bloodstream. Therefore, this perspective will facilitate the essential development required to successfully manufacture effective theranostic NPs for human health care.

### Conflict of interest statement

The authors declare that the research was conducted in the absence of any commercial or financial relationships that could be construed as a potential conflict of interest.
